# The Cellular Accumulation of Vehicle Exhaust Particulates Changes the Acidic pH Environment of Lysosomes in BEAS-2B Airway Epithelial Cells

**DOI:** 10.3390/jox13040042

**Published:** 2023-11-01

**Authors:** Akira Onodera, Takuya Shimomura, Hirohisa Ochi, Ryuto Sunada, Eiko Fukutomi, Koushi Hidaka, Yuichi Kawai

**Affiliations:** 1 Department of Pharmaceutical Sciences, Kobe Gakuin University, 1-1-3 Minatojima, Chuo-ku, Kobe 650-8586, Japan; u510276j@ecs.osaka-u.ac.jp (T.S.); hirohisaob@outlook.jp (H.O.); ryuto.sunada.ye@kyowakirin.com (R.S.); yingzifufu@gmail.com (E.F.); pa105292@pharm.kobegakuin.ac.jp (Y.K.); 2 Research Facility Center for Science and Technology, Kobe University, 1-1, Rokkodai-cho, Nada-ku, Kobe 657-8501, Japan; khidaka@people.kobe-u.ac.jp

**Keywords:** PM2.5, vehicle exhaust particulates, lysosome, extracellular vesicles

## Abstract

Many people are exposed every day to vehicle exhaust particulates (VEPs), which are thought to be taken up by epithelial cells that are the first barrier in our biological defense. The study aim was to investigate how VEPs are processed in the lysosomal degradation system. BEAS-2B airway epithelial cells easily ingest VEPs and have been shown to accumulate in cells for several days, but no elevated cytotoxicity was observed over that time period. An analysis of 3D images confirmed the presence of VEPs in or near lysosomes, and an accumulation of VEPs resulted in an increase in the normal acidic pH in lysosomes and the extracellular release of the lysosomal enzyme β-hexosaminidase. Epithelial cells were thought to activate the lysosome-mediated secretion of extracellular vesicles to avoid damage caused by non-degradable foreign substances, such as VEPs, and as a side reaction, the acidic pH environment of the lysosomes could not be maintained.

## 1. Introduction

Environmental xenobiotics include suspended particulate matter (SPM) that binds to cell surfaces and is progressively enclosed by a part of the cell membrane, which invaginates and then pinches off to form a small vesicle containing the xenobiotic. This process is called endocytosis, which is classified roughly into two types according to the size of the endocytic vesicles: pinocytosis, that forms small 100 nm diameter vesicles, and phagocytosis that forms large ≥250 nm diameter vesicles. It is thought that SPM is also processed as a xenobiotic by endocytosis [1].

Particulate matter with an aerodynamic diameter of ≤2.5 μm (PM2.5) is considered to be one cause of inflammation by SPM. The inflammatory mediator of IL-8 is induced by PM2.5 but decreased by the endocytosis inhibitor cytochalasin D in the airway cells of human bronchial epithelial and macrophage-like cell lines by BEAS-2B and THP-1, respectively [2].

Prior to endocytosis, SPM is delivered to an endosome compartment called an early endosome. Some of the receptors in early endosomes are recycled back to the plasma membrane via transport vesicles. SPM should not be retrieved from early endosomes but instead be delivered to lysosomes. The lysosomal lumen is maintained at approximately pH 4.5–5 and contains 40 degradative enzymes that are active in this environment. The maintenance of lysosome function is important for degrading xenobiotics and recycling misfolded proteins and damaged organelles [3]. The disruption of lysosome function is critical for the pathogenesis and development of various diseases [4,5,6]. The accumulation of monosodium urate crystals in lysosomes is considered a cause of lysosomal membrane damage and subsequently induces the secretion of pro-inflammatory cytokines [7,8]. This mechanism is related to the activation of the NOD-like receptor family pyrin domain containing three (NLRP3) inflammasomes, which is also activated by PM2.5 [9,10]. A broncho-alveolar lavage of patients with chronic obstructive pulmonary disease has shown high levels of interleukin-1-like cytokines, the induction of which is related to the NLRP3 inflammasome [11]. A growing body of evidence has shown that NLRP3 inflammasomes are critically involved in the pathogenesis of severe asthma [12].

The disruption of lysosome function not only causes inflammation, but also poses a serious threat to cells via apoptosis and necroptosis [13]. The cholinesterase inhibitor of tacrine induces lysosomal membrane permeabilization and causes cathepsin B leakage from lysosomes [14]. Cathepsin B, a lysosomal cysteine protease, cleaves apoptosis-regulating bcl-2 family proteins, leading to Bax/Bak-mediated mitochondrial membrane permeabilization and the release of cytochrome c from mitochondria, which is a major event during apoptosis [15]. The polyinosinic–polycytidylic acid sodium salt, as a synthetic analog of double-stranded RNA, induces a loss of lysosome integrity, leading to necroptosis in a cathepsin-D-dependent manner [16].

Lysosomes degrade and recycle intracellular and extracellular substances, but they also function as organelles that are susceptible to damage from foreign substances. It is unclear how lysosomes handle foreign substance, such as PM2.5, that are considered difficult to degrade by lysosomal enzymes. The study aim was to investigate how vehicle exhaust particulates (VEPs), one of the sources of PM2.5, are taken up by cells in humans and processed by the lysosomal degradation system. Our results showed that VEP_S_ were easily and abundantly taken up by cells and transported to lysosomes in cultured cells. The intracellular accumulation of VEPs reduced the pH stability of lysosomes through extracellular vesicle secretion via lysosomes.

## 2. Materials and Methods

### 2.1. Cell Culture

The human airway epithelium BEAS-2B cell line was obtained from the American Type Culture Collection (Manassas, VA, USA) and cultured in complete media consisting of DMEM/Ham’s F-12 (Nacalai Tesque, Kobe, Japan) supplemented with 0.1% or 10% bovine growth serum (Cytiva, Tokyo, Japan) and antibiotic-antimycotic (Nacalai Tesque) in a humidified CO_2_ incubator at 37 °C. Before using BEAS-2B for various experiments, cells were proliferated in a state of sub-confluent growth by subculturing with trypsin three times every 3 days for the enrichment of BEAS-2B.

### 2.2. Model Materials of SPM

VEPs were distributed by the National Institute for Environmental Studies in Japan (CRM No.8). The origin of VEPs was atmospheric particulate matter collected with electrostatic precipitators in a highway tunnel in Japan. VEPs were suspended at 5 mg/mL in Dulbecco’s phosphate-buffered saline and sonicated for 30 min prior to use.

### 2.3. Lactate Dehydrogenase Cytotoxicity Assay

Cytotoxicity was analyzed by measuring the leakage of lactate dehydrogenase (LDH) into the cell culture supernatant. BEAS-2B cells were seeded for 24 h in a 12-well plate (Greiner Bio-One, Kremsmünster, Austria) at a density of 1 × 10^5^ cells/well. Cells were pretreated with specific vacuolar-type H+-ATPase inhibitor of bafilomycin A1 (BafA1) or vehicle for 2 h and then treated with 0, 7.4, 22.2, 66.7, and 200 µg/mL of VEPs. After 24 h, all of the cell culture supernatant was collected from each well, and new culture media was added. After 96 h, all of the cell culture supernatant was collected again. The amount of LDH was measured using an LDH-Cytotoxic Test Wako (Fujifilm Wako Pure Chemical Co., Osaka, Japan), according to the manufacturer’s instructions.

### 2.4. Intracellular Dynamics of VEPs

The time taken for VEPs to be taken up by cells was measured by observing under a FV3000 Confocal Laser Scanning Microscope (CLSM; Olympus, Tokyo, Japan). BEAS-2B cells were seeded for 24 h in a glass-bottomed dish (Ibidi GmbH, Martinsried, Germany) at a density of 3 × 10^4^ cells/well. Then, the cells were treated with 50 or 200 µg/mL of VEPs. After incubation at 37 °C for 0, 6, and 24 h, the CLSM images were captured. Subsequently, the residual VEPs in the culture media were removed, together with the culture supernatant, and new culture media was added to continue the cell culture. After 24, 48, and 72 h, or only 48 h, CLSM images were captured.

Using 3D refractive index imaging and 2D imaging based on holograph and tomography technologies, the 3D Cell Explorer (Nanolive, San Francisco, CA, USA), a holographic microscope, can investigate cells and their internal structures in 3D without the need for staining chemicals. To assess the intracellular distribution of VEPs, cell membrane refractive indices, as well as VEPs-specific refractive indices, were digitally dyed in yellow and red, respectively, for BEAS-2B cells 3 h after VEP treatment.

### 2.5. Lysosomal Acidification Stability

Acridine orange (AO; Sigma-Aldrich, Tokyo, Japan), a metachromatic fluorescent dye, accumulates in lysosomes via proton trapping and was used to evaluate the lysosomal acidification stability. We analyzed the effect of VEPs on lysosomal acidification stability using fluorescence imaging of AO. BEAS-2B cells were seeded for 24 h in a glass-bottomed dish at a density of 3 × 10^4^ cells/well. The cells were treated with 50 µg/mL of VEPs. After 24 h, the cells were washed with complete media three times. After that, or 72 h later, 1 µg/mL of AO was loaded into cells that were then incubated at 37 °C for 10 min. Live-cell AO imaging was performed using an FV3000, and the images were analyzed using FV31S-SW viewer software (Olympus, Tokyo, Japan). AO in acidic lysosomes was excited at 488 nm, and the emission at 617–673 nm was detected. A lysosome deacidified by treatment with 50 nM BafA1 for 1 h was prepared as a negative control.

### 2.6. Beta Hexosaminidase (β-HEX) Activity Assay

β-HEX is a hydrolase in lysosomes that is released from cells by the secretion of extracellular vesicles. We measured β-HEX activity using p-nitrophenyl N-acetyl-ß-D-glucosaminide (pNAG) as a substrate, which produces p-nitrophenol with maximum absorption at 405 nm following β-HEX hydrolysis. BEAS-2B cells were seeded for 24 h in a 48-well plate at a density of 1 × 10^5^ cells/well. The cells were pretreated with BafA1 or vehicle for 2 h and then treated with 0, 5, and 50 µg/mL of VEPs for 24 h. After treatment, the cell culture supernatants were collected and incubated with 5 mM pNAG (Nacalai Tesque) in 26 mM citrate buffer, pH 4.5, for 4 h at 37 °C. The reaction was quenched by adding glycin buffer, pH10. Absorbance at 405 nm (570 nm reference absorbance) was determined using a SYNERGY LX microplate reader (BioTek, Tokyo, Japan).

### 2.7. Statistical Analysis

All assays were performed in triplicate, at least. The analysis of the data was performed using ANOVA and Tukey’s HSD tests with Kaleida Graph statistical software (HULINKS, Tokyo, Japan).

## 3. Results

### 3.1. Cytotoxicity of VEPs

**Figure 1:**
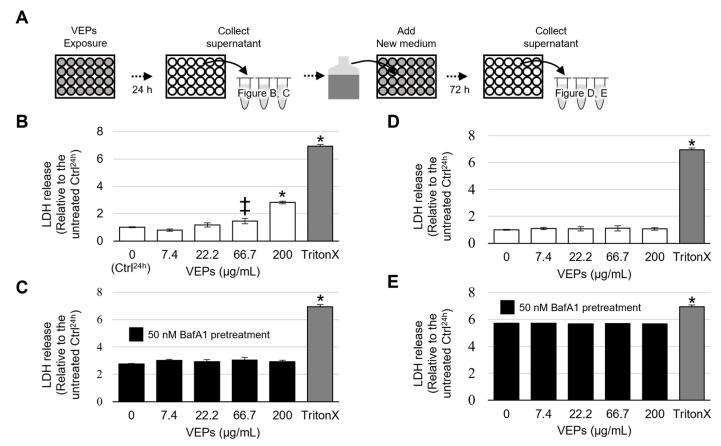
**Effect of VEPs’ accumulation and deacidification by BafA1 on VEPs’ cytotoxicity.** Collection of culture supernatants and treatment of VEPs for use in the LDH assay (**A**). LDH assay result calculation of relative 0 µg/mL of VEPs for 24 h (Ctrl24). A cell lysate permeabilized with Triton X was used as a positive control. In this analysis, we evaluated the effects of VEPs treatment for 24 h (**B**,**C**) and after the next 72 h (**D**,**E**), as well as the effects of VEPs on lysosomal deacidification by BafA1 (**C**,**E**). * *p* < 0.001 vs. Ctrl^24h^; ‡ *p* < 0.05 vs. Ctrl^24h^.

LDH is present in the cytoplasm and leaks into the culture media because of cell membrane damage. The 24 h treatment of BEAS-2B cells with VEPs significantly increased the leakage of LDH into the culture media at 66.7 and 200 µg/mL (Figure 1A,B). As shown in Figure 1A, BEAS-2B was treated with VEPs for 24 h and then cultured continuously for 72 h, but there was no significant increase in LDH leakage even at a concentration of 200 µg/mL (Figure 1A,C). To suppress the acidification (deoxidation) of lysosomal lumen, BafA1 was added to the culture media before VEPs treatment for 1 h. Compared to the 24 h control (VEPs and BafA1 untreated cell), the leakage of LDH was increased by BafA1 regardless of VEPs treatment. In contrast, no future increases or enhancements of the amount of leakage of LDH by VEPs was confirmed (Figure 1A,D). As a side note, continuous lysosomal deoxidation with BafA1 treatment for a total of 96 h killed all cells regardless of VEPs treatment, as did the treatment of cell membrane permeation with a surfactant (Figure 1A,E).

### 3.2. Chronological Observation of VEPs’ Ingestion and Evacuation

**Figure 2:**
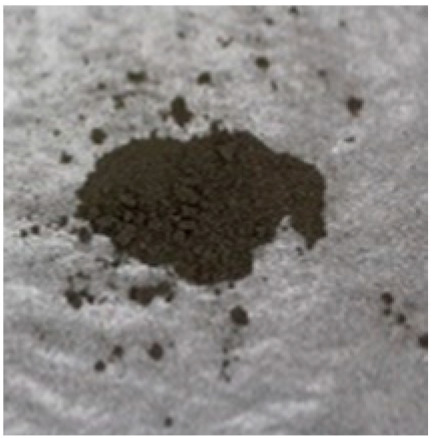
**Form of VEPs as powder.** The VEPs provided by the National Institute for Environmental Studies, Japan, were placed on packaging paper, and the powder state was photographed and imaged with a digital single-lens reflex camera (Canon, Tokyo, Japan).

The intracellular behavior of VEPs can be easily observed under an optical microscope, since VEPs are black (Figure 2).

**Figure 3:**
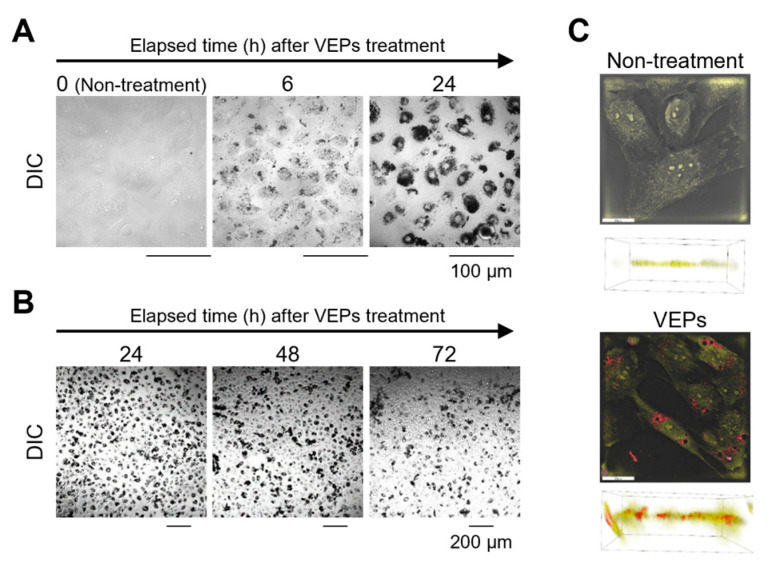
**Time required for the intracellular ingestion of VEPs.** To determine how long it takes for VEPs to be ingested into cells, the cells were imaged on a confocal laser scanning microscope (FLUOVIEW FV3000) at 6 h and 24 h after VEPs treatment (**A**). To determine how long the VEPs remained in the cells, the VEPs were treated for 24 h and the cells were imaged after the next 24, 48, and 72 h (**B**). To clarify the intracellular localization of VEPs, the VEPs were treated for 3 h and the cells were digitally dyed in yellow and red, respectively, and imaged (**C**).

When 100 µg/mL of VEPs were added to BEAS-2B cells, the clear intracellular ingestion of VEPs was observed under an optical microscope after 6 h. After the next 18 h, VEPs accumulated throughout the cytoplasm (Figure 3A). The cellular components and VEPs were digitally stained in yellow and red, respectively, using 3D Cell Explorer, and the intracellular localization of VEPs was observed (Figure 3B). The monitoring of VEPs ingested by cells every 24 h showed a decrease in VEPs’ accumulation after 48 h and an additional decrease after 72 h (Figure 3C). However, the degree of VEPs accumulation and subsequent reduction were different for each individual cell. A large number of cells were observed to continue accumulating VEPs.

### 3.3. Lysosomes Were Deoxidized by the Accumulation of VEPs

**Figure 4:**
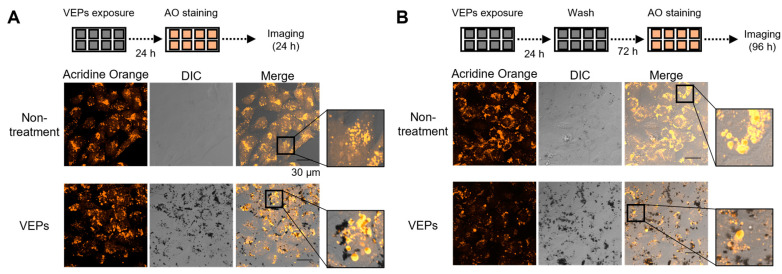
**Induction of lysosomal deacidification by VEPs.** To observe the state of intracellular acidic compartments, the cells were stained with AO and imaged with FV3000 at 24 h and 72 h after VEPs treatment (**A**,**B**).

We performed an AO staining procedure to determine whether the acidic pH in lysosomes was maintained in the cells that accumulated VEPs. AO is a double-fluorescent dye that binds to nucleic acids and enters acidic sites, such as lysosomes, and has different maximum excitation and emission wavelengths (Ex/Em max). The Ex/Em max of AO in lysosomes is 460 nm/650 nm, and the fluorescence intensity indicates that the acidic environment of lysosomes is maintained. In BEAS-2B cells 48 h after seeding on a slide glass, strong AO fluorescence was observed in the cytoplasmic region (Figure 4A,B). After 24 h VEPs treatment, the fluorescence image of AO was similar to that of the non-treatment at first glance, but when the area with strong AO fluorescence was enlarged, vesicles similar to blebbing were observed, and the AO fluorescence in the cytoplasm was weak (Figure 4A,B). In BEAS-2B cells 120 h after seeding on a slide glass, the AO fluorescence in the cytoplasm was weaker than in the non-treatment (Figure 4A), and the AO fluorescence from vesicles was similar to that of the 24 h VEPs treatment cells (Figure 4A,C). After culturing for 72 h with accumulated VEPs, the AO fluorescence in the cytoplasm was greatly reduced (Figure 4B).

### 3.4. VEPs Promote the Release of β HEX

**Figure 5:**
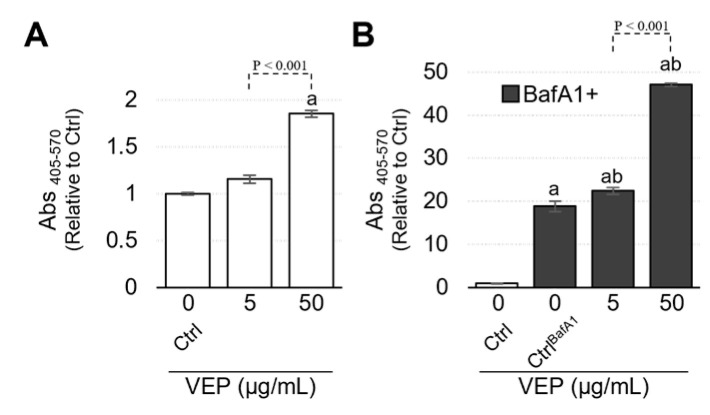
**Increased β-HEX release by VEPs.** To clarify the extracellular vesicles’ release from lysosomes by VEPs, the extracellular release of the lysosomal enzyme β-HEX was measured. β-HEX assay result calculation of relative 0 µg/mL of VEPs for 24 h (Ctrl). In this analysis, we evaluated the effects of VEPs treatment for 24 h (**A**) and the effects of VEPs on lysosomal deacidification by BafA1 (**B**). a: *p* < 0.001 vs. Ctrl; b: *p* < 0.001 vs. Ctrl^BafA1^.

The lysosomal enzyme β-HEX is widely used as an indicator of extracellular vesicle secretion via lysosomes. Lysosome-mediated pathways are known to be one of the origins of exocytosis, thought to be due to proton release within lysosomes [17]. Cell culture supernatants treated with VEPs for 24 h were collected and measured for B-HEX activity, which was significantly increased at 50 µg/mL (Figure 5A). Treatment with BafA1 significantly increased the β-HEX activity regardless of VEPs treatment (Figure 5B). Co-treatment with VEPs and BafA1 significantly increased β-HEX activity even at 5 µg/mL, and increased it further at 50 µg/mL VEPs (Figure 5B).

## 4. Discussion

**Figure 6:**
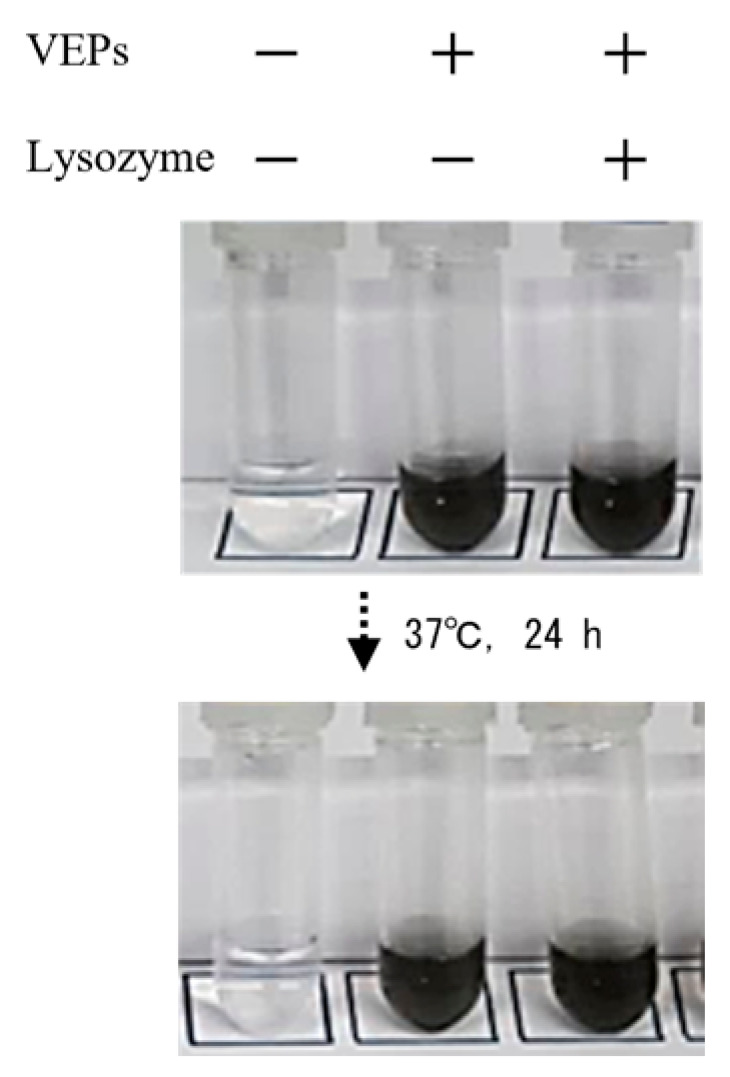
**Form of VEPs in lysozyme solution.** To clarify the solubility of VEPs with enzymatic treatment, VEPs were suspended in 100 µg/mL human lysozyme (Sigma-Aldrich, Tokyo, Japan) solution in 10 mM citrate buffer (pH 5.0) and incubated at 37 °C. After 24 h, the VEPs were resuspended by vertexing and photographed and imaged with a DLSR camera.

The intracellular accumulation of SPM is thought to be one of the major factors in the onset of toxicity, and an increase in the amount or duration of SPM accumulation is even thought to exacerbate cytotoxicity [18]. The SPM model VEPs used in this study were easily taken up by cells, but cytotoxicity was weak and observed as a transient phenomenon in the initial period of exposure (24 h after). The accumulation of intracellular VEPs did not exhibit subsequent cytotoxicity (Figure 1B,C). VEPs were not degraded even when treated with acidic or neutral solutions or with lysozyme, which is responsible for innate immunity lysis (Figure 6).

The intracellular behavior of extracellular foreign substances depends on endosomes and is degraded by fusion with lysosomes. However, VEPs are thought to maintain a very stable structure within the cell. Three-dimensional cell images showed the accumulation of VEPs in vesicles that were thought to be lysosomes (Figure 3C). On the other hand, it is thought that the degradation of VEPs, which act like a lump of carbon, by lysosomal enzymes responsible for the degradation of biological components is impossible. After AO staining of VEPs-accumulating cells, the fluorescence (Ex/Em: 488 nm/617–673 nm) emitted in an acidic environment was attenuated, indicating that the acidification of the lysosomal internal environment was not maintained. Many secretory vesicles with a diameter of approximately 0.3 µm were formed from these cells, and a high fluorescence of AO was observed (Figure 4). In PM cytotoxicity, the maintenance of the acidification of the lysosomal lumen has contradictory roles of avoidance or the causation of cytotoxicity. The use of BafA1 inhibited the acidification of the lysosomal lumen, and the cytotoxicity of cobalt nanoparticles and copper oxide nano- and microparticles decreased, whereas that of silver nanoparticles increased [19,20,21]. In this study, pre-treatment with BafA1 reduced the cytotoxicity of VEPs, and the inhibition of the acidification of the lysosomal lumen by VEPs accumulation is thought to prevent subsequent cytotoxicity.

Exosomes with 40–120 nm diameter and micro-vesicles with 50–1000 nm diameter are secretory vesicles formed by exocytosis via one of the intercellular communication mechanisms [22]. Although lysosomes are the source of secretory vesicles, the formation mechanism and triggers are unclear [18]. Rat alveolar epithelial cells activate lysosome-derived exocytosis in the presence of polystyrene nanoparticles and are thought to be associated with lung epithelial injury that causes interstitial lung disease [23]. Mesoporous silica also activates lysosome-derived exocytosis, but deacidification by BafA1 reduces it and exacerbates cytotoxicity [24]. In the case of nanoparticles, which have high catalytic activity in biological components, the role played by lysosome-derived exocytosis is thought to exacerbate injury.

The main element of black VEPs is thought to be carbon and its compounds, polycyclic aromatic hydrocarbons. In addition, VEPs include >30 types of elements (VEP elements data: https://www.nies.go.jp/labo/crm-e/index.html accessed on 24 October 2023), and particles containing these elements are observed as stable aggregates within cells and lysosomes (Figure 3). Similar to the turnover in other organelles, the turnover in lysosomes to maintain their quality is regulated by autophagy, even in normal lysosomes that have regular metabolic cycles [25,26]. It is thought that lysosomes that have accumulated nondegradable foreign substances, such as VEPs, cannot participate in these metabolic cycles, and to maintain lysosome function, extracellular vesicles release nondegradable foreign substances. In lysosomal storage diseases caused by excessive lipid accumulation, exocytosis from lysosomes is thought to be effective in improving the pathology, and these mechanisms are considered to be among various mechanisms for eliminating foreign substances [27].

BafA1 treatment results in the excess secretion of β-HEX, suggesting the pH-dependent regulation of secretory vesicle formation from lysosomes and a sensor molecule that monitors the acidification of the lysosomal lumen according to its state of charge. Keap-1, a sensor molecule for oxidative stress, recognizes changes in the charge state of amino acids as one of its recognition mechanisms [28]. As the pH of the solution changes from acidic to neutral, the charge state of proteins and amino acids changes from positive to negative. Although the significance of extracellular vesicle release from lysosomes due to the intracellular accumulation of VEPs and pH changes is unclear, the elimination of foreign substances that cannot be degraded by lysosomes is thought to help prevent cytotoxicity.

## 5. Conclusions

Technological improvements in various industries have reduced the emissions of hazardous compounds [29,30,31]. On the other hand, SPM that threatens our health can easily cross national borders, and the number of allergic respiratory diseases and heart diseases which are thought to be caused by SPM in the air has not decreased. The VEPs used in this report were thought to be those that people are exposed to daily in Japan. Although VEPs are not highly cytotoxic, they are easily taken up by cells and continue to accumulate for several days. VEPs that have entered the body are thought to be actively taken up by phagocytic cells, such as macrophages and dendritic cells, and these cells continue to survive in the body for several days while accumulating VEPs. Inside these cells, VEPs are transported into lysosomes and subsequently inhibit the maintenance of the acidic environment of lysosomes, resulting in decreases in immune responses and in the recycling function in which lysosomes act as reaction sites. The lysosome-mediated secretion of extracellular vesicles is thought to prevent these VEPs effects. Further studies that elucidate these mechanisms and their physiological roles in vivo would be useful.

## Data Availability

The data presented in this study are available on request from the corresponding author.
